# Discrete False-Discovery Rate Improves Identification of Differentially Abundant Microbes

**DOI:** 10.1128/mSystems.00092-17

**Published:** 2017-11-21

**Authors:** Lingjing Jiang, Amnon Amir, James T. Morton, Ruth Heller, Ery Arias-Castro, Rob Knight

**Affiliations:** a Department of Pediatrics, University of California San Diego, La Jolla, California, USA; b Department of Family Medicine and Public Health, University of California San Diego, La Jolla, California, USA; c Department of Computer Science and Engineering, University of California San Diego, La Jolla, California, USA; d Department of Statistics and Operations Research, Sackler Faculty of Exact Sciences, Tel Aviv University, Tel Aviv, Israel; e Department of Mathematics, University of California San Diego, La Jolla, California, USA; f Center for Microbiome Innovation, University of California San Diego, La Jolla, California, USA; University of Waterloo

**Keywords:** differential abundance, discrete test statistics, FDR, high dimension, microbiome, multiple comparison, multiple testing, sparse, statistics

## Abstract

Differential abundance testing is a critical task in microbiome studies that is complicated by the sparsity of data matrices. Here we adapt for microbiome studies a solution from the field of gene expression analysis to produce a new method, discrete false-discovery rate (DS-FDR), that greatly improves the power to detect differential taxa by exploiting the discreteness of the data. Additionally, DS-FDR is relatively robust to the number of noninformative features, and thus removes the problem of filtering taxonomy tables by an arbitrary abundance threshold. We show by using a combination of simulations and reanalysis of nine real-world microbiome data sets that this new method outperforms existing methods at the differential abundance testing task, producing a false-discovery rate that is up to threefold more accurate, and halves the number of samples required to find a given difference (thus increasing the efficiency of microbiome experiments considerably). We therefore expect DS-FDR to be widely applied in microbiome studies.

**IMPORTANCE** DS-FDR can achieve higher statistical power to detect significant findings in sparse and noisy microbiome data compared to the commonly used Benjamini-Hochberg procedure and other FDR-controlling procedures.

## Abstract (precis)

DS-FDR can achieve higher statistical power to detect significant findings in sparse and noisy microbiome data compared to the commonly used Benjamini-Hochberg procedure and other FDR-controlling procedures.

## INTRODUCTION

An important goal of many microbiome analyses is to identify key microbes that explain differences between groups of samples. This differential abundance testing is used in numerous applications, such as pinpointing pathogens and beneficial agents that differentiate healthy and disease states (1). This problem is complicated by the fact that microbial communities are extremely complex, with as many as thousands of species within samples, leading to the need for multiple-hypothesis correction.

In the era of big data, and especially in microbiome studies, adjusting for multiple-hypothesis testing is necessary. The multiplicity problem was first encountered in highly multivariate data sets in the social sciences, and it was regarded by Wilkinson as the “curse of social sciences” (2). This is because the probability of a type I error (a single hypothesis incorrectly yielding a positive result) increases dramatically with the large numbers of hypotheses. Performing differential abundance analysis in microbiome studies often requires thousands of multiple-hypothesis tests, greatly increasing the risk of misidentifying differentially abundant microbes.

**FIG 1:**
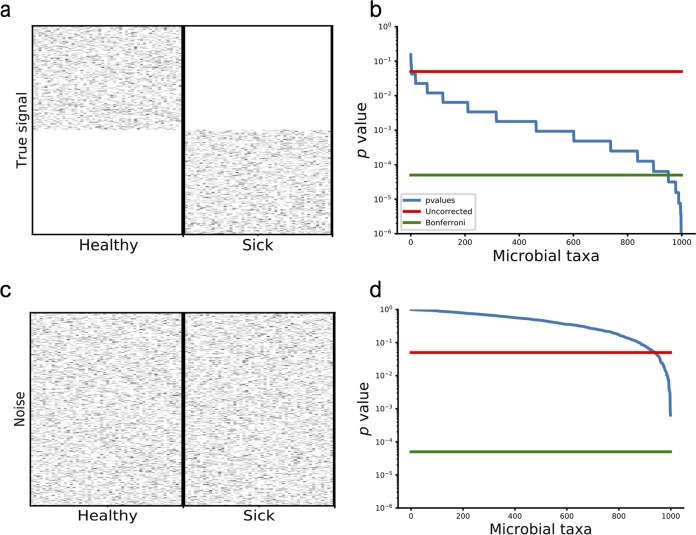
Illustration of the multiple-hypothesis testing problem. (a) A heatmap of the simulated microbial abundances with 1,000 differentially abundant microbes from 50 sick patients and 50 healthy patients. (b) The sorted *P* values for the microbes shown in panel a from a Mann-Whitney U test. (c) A heatmap of the randomly simulated abundances with 1,000 microbes and 100 samples. (d) The sorted *P* values for each of the microbes from a Mann-Whitney U test from panel c.

To better understand the multiple-comparison problem, consider the following two scenarios. In Fig. 1a, we simulate two groups of samples collected from a sick cohort and a healthy cohort. In this case, the signal is extremely obvious—the healthy control cohort has a completely different set of microbes compared to the sick cohort. This difference is so strong that the healthy and sick cohorts have no common microbes. According to the *P* values from the Mann-Whitney U tests, the vast majority of the microbes have a *P* value of less than 0.05 (Fig. 1b). In Fig. 1c, there are no signals that differentiate sick and healthy patients, but there are still about 50 microbes that have *P* values of less than 0.05 (Fig. 1d) according to individual Mann-Whitney U tests. These *P* values do not reflect a true signal differentiating the sick and healthy patients, and in this case we know they arose purely by chance in the simulations. This is not only apparent for the Mann-Whitney U test but for any statistical test.

One of the underlying issues is the inflated type I errors. Multiple testing correction is used to obtain thresholds that lower the inflated type I, or false-positive, errors. Bonferroni’s correction is a simple and popular method to control the family-wise error rate (FWER), or the probability that at least one test out of the entire set will incorrectly yield a positive result. Bonferroni’s correction provides strong control of type I errors (incorrectly rejecting the null hypothesis when it is true) but often results in a high rate of type II, or false-negative, errors (incorrectly accepting the null hypothesis when it is false). As a result, the power of the study sharply decreases as the number of tests increases. This is especially a problem for microbiome studies, because the strict FWER thresholds required to reject the null hypothesis for each microbe becomes extremely small, thus erasing evidence of many differentially abundant taxa and incorrectly leading to conclusions that there are no differences in the microbiome. As shown in Fig. 1d, Bonferroni’s correction may guard against false-positive errors, but in Fig. 1b, the same Bonferroni’s correction will miss many differentially abundant species.

Especially in early stages of analysis, it is often preferable to tolerate false-positive results in order to identify interesting cases that would generate hypotheses for further investigation, as long as the proportion of false-positive results is maintained at a relatively low and well-defined level. Consequently, in microbiome applications, it is preferable to apply a multiple-comparison procedure that controls the false-discovery rate (FDR), defined as the expected proportion of false-positive findings among all the findings (i.e., rejected hypotheses) (3). The procedure introduced by Benjamini and Hochberg (3) (henceforth, the BH procedure) is the first multiple testing procedure for controlling the FDR and remains very popular. For independent continuous test statistics, the FDR of the BH procedure at level *q* (controlled level of false-discovery rate) is exactly $\frac{m_{0}}{m}q$, where *m* and *m*_0_ are the number of hypotheses and the number of true null hypotheses, respectively (3). However, for discrete test statistics, as their tail probabilities can be smaller than those of continuous test statistics from the null distribution, the FDR of the BH procedure may be much smaller than $\frac{m_{0}}{m}q$, resulting in overconservative control of the FDR and reduced power in detecting significant findings (4).

Two features of microbiome data could easily produce discrete test statistics and possibly highly conservative FDR control: one is the low number of samples, and the other is the underlying sparsity of the data. The typical number of samples in a microbiome experiment ranges from ten to a few thousand, and when the sample sizes are around ten, discreteness becomes a concern. Sparsity (the proportion of nonzero values in the data) is typically on the order of 1% to 10%, indicating that most taxa are present only in a very small number of samples, and this leads to discreteness of the resulting test statistics and overconservative FDR control. To overcome this conservatism, we introduce the discrete FDR (DS-FDR) method. This procedure coincides with the permutation-based FDR estimation procedure of Li and Tibshirani (5). By permuting the labels, this nonparametric method exploits the discreteness of the test statistics and achieves better power than the BH procedure. It also achieves better power than the procedure that first filters out hypotheses that have no power to be rejected at the nominal level, the filtered BH (FBH) method (which is similar to one of the variants suggested in reference 6). We demonstrate in both simulations and experimental microbiome data that DS-FDR is able to detect a larger number of significant findings than the BH and FBH procedures under the same FDR control level or, conversely, detect the same findings using a smaller sample size.

## RESULTS AND DISCUSSION

### Simulations.

The ideal FDR control method should return the maximal number of significant taxa, while returning as few falsely identified taxa as possible. However, in most cases, removing false-negative results also results in removal of more true-negative results (since we are decreasing the *P* value threshold), leading to a type I/type II error balance. Given that the tolerable FDR level is defined by the researcher, we therefore measure the performance of the FDR methods by comparing the number of true significant taxa detected, as long as the FDR is controlled at or below the desired level.

To test the validity and performance of the FDR control methods, we first used simulated communities, which provide a known ground truth, and enable changing relevant parameters to test the effect on the performance of the method. Simulations were composed of two groups (sick and healthy groups), each composed of multiple samples drawn from a multinomial distribution of the group. In each simulation, 100 taxa were drawn from a different distribution between the sick and healthy groups (i.e., truly different), an additional 100 originating from the same distribution (i.e., not different), and the rest were introduced as a set of rare taxa, which were the same across groups yet present in a very small number of samples (see Text S1 and Table S1 in the supplemental material for simulation details). Unless otherwise stated, in the following simulations, sample size varies from 10 to 100 per group, the difference of the mean rank is used as the test statistic, *q* = 0.1 is chosen as the threshold for FDR, and 1,000 permutations are performed in FDR calculation.

10.1128/mSystems.00092-17.1 TEXT S1  The supplemental text file includes simulation details, data-driven simulation details, and proof of the equivalence of the DS-FDR to Heyse’s DBH procedure in permutation. Download TEXT S1, DOCX file, 0.1 MB. Copyright © 2017 Jiang et al. 2017 Jiang et al. This content is distributed under the terms of the Creative Commons Attribution 4.0 International license.

10.1128/mSystems.00092-17.4 TABLE S1  Setting of simulated microbiome communities. Download TABLE S1, DOCX file, 0.04 MB. Copyright © 2017 Jiang et al. 2017 Jiang et al. This content is distributed under the terms of the Creative Commons Attribution 4.0 International license.

**FIG 2:**
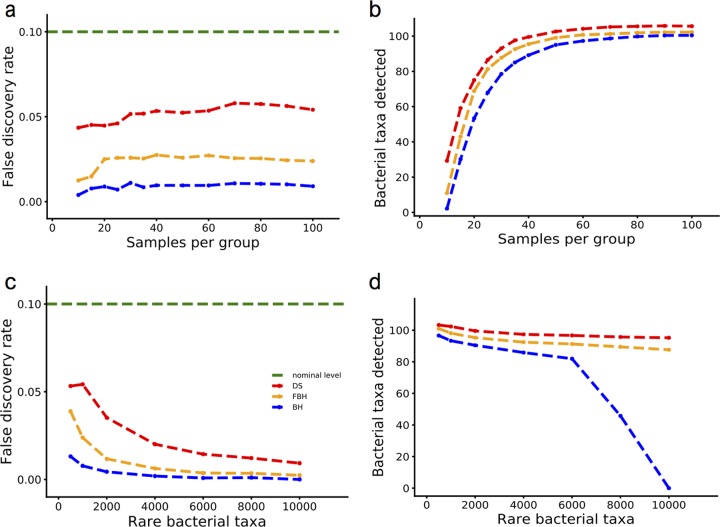
Comparison of FDR methods on simulated data sets. (a) Average FDR for the DS, FBH, and BH procedures (red, orange, and blue lines, respectively) as a function of the number of samples per group in simulation I. The green line indicates the prespecified FDR control level (0.1) used for the tests. (b) Number of differentially abundant taxa identified by the three methods in panel a with 100 truly different taxa and 900 truly nondifferent taxa. (c) Average FDR for DS, FBH, and BH procedures as a function of the number of rare taxa in simulation II (50 samples per group). The green line indicates the prespecified FDR control level (0.1) used for the tests. (d) Number of differentially abundant taxa identified by the three methods in panel c.

In the first simulation, we test the effect of the number of samples on the performance of the Benjamini-Hochberg (BH) procedure, the proposed filtered BH (FBH), and DS-FDR method (Fig. 2a and b). Specifically, we simulate 100 truly different taxa, 100 taxa coming from the same distribution, and 800 rare taxa coming from the same distribution. While all methods safely control the FDR at the desired level (less than 10%), the BH procedure is the most conservative, obtaining an FDR of ~1% (compared to ~2.5% in FBH and ~5% in DS-FDR) (Fig. 2a). As expected by the lower conservatism, DS-FDR yields increased power to detect statistically significantly differing taxa. As shown in Fig. 2b, DS-FDR outperforms FBH and BH in identifying the differentially abundant taxa between two groups despite changes in the sample size: on average, DS-FDR is able to detect at least 15 more significant taxa than BH and 6 more than FBH (recall that the total number of truly differing taxa in these simulations is 100). Moreover, the advantage of DS-FDR over the other two procedures is greater when the sample size is small. For example, when the sample size is at most 20, DS-FDR identifies 24 more taxa than BH, and 16 more than FBH, on average. This is because smaller sample size leads to more discrete test statistics (see Fig. S1 in the supplemental material). Therefore, under these simulated conditions, DS-FDR has more power to detect significant taxa than BH and FBH, while also controlling the FDR under the desired level.

10.1128/mSystems.00092-17.2 FIG S1  Discreteness of test statistics revealed in *P* value distributions in simulation 1. The green, purple, and blue lines represent the cumulative distribution of unadjusted *P* values at sample sizes of 50, 15, and 10, respectively. The red line represents the uniform distribution of *P* values. Download FIG S1, TIF file, 2.6 MB. Copyright © 2017 Jiang et al. 2017 Jiang et al. This content is distributed under the terms of the Creative Commons Attribution 4.0 International license.

In the second simulation, we increase the sample size to 50 per group to alleviate the discrete effect caused by small sample size but add more rare taxa (500 to 10,000 more rare taxa) to check the effect of discreteness resulting from sparsity. This is a relevant question for microbiome analysis, because sparse abundance matrices are common in microbiome experiments, due to both noise in the OTU (operational taxonomic unit) picking method and the real low-abundance taxa. Figure 2c and d show the effects of such sparse taxa on the performance of the different FDR methods. As in the previous simulation, BH is the most conservative, with the lowest FDR dropping to almost 0% when the number of rare taxa increases to 10,000. Affected by such a highly conservative FDR control, the number of significant taxa that BH can detect is always lower than the numbers detected by DS-FDR and FBH, and its deteriorating performance becomes most obvious when the number of rare taxa goes beyond 8,000 (Fig. 1d). In contrast, FBH and DS-FDR yield stable performance in detecting differentially abundant taxa despite the increasing noise, while controlling the FDR below the threshold, with DS-FDR consistently finding more taxa than FBH. This shows that DS-FDR has an advantage in finding differentially abundant taxa in sparse data. This is due to the fact that the sparser the data, the more severe the discreteness problem, and thus the more power DS-FDR gains (Fig. S2).

10.1128/mSystems.00092-17.3 FIG S2  Discreteness of test statistics revealed in *P* value distributions in simulation 2. The green, blue, and orange lines represent the cumulative distribution of unadjusted *P* values at sample sizes of 500, 2,000, and 6,000, respectively. The red line represents the uniform distribution of *P* values. Download FIG S2, TIF file, 2.6 MB. Copyright © 2017 Jiang et al. 2017 Jiang et al. This content is distributed under the terms of the Creative Commons Attribution 4.0 International license.

### Data-driven simulations.

To provide more-comprehensive simulations using real-microbiome read distributions, we generated data-driven simulations based on the following two real-microbiome data sets: DIBD (gut microbiome in dog and human inflammatory bowel disease) (7) and CS (microbial communities in the upper respiratory tract of cigarette smokers) (8) (see Text S1 for data-driven simulation details).

**FIG 3:**
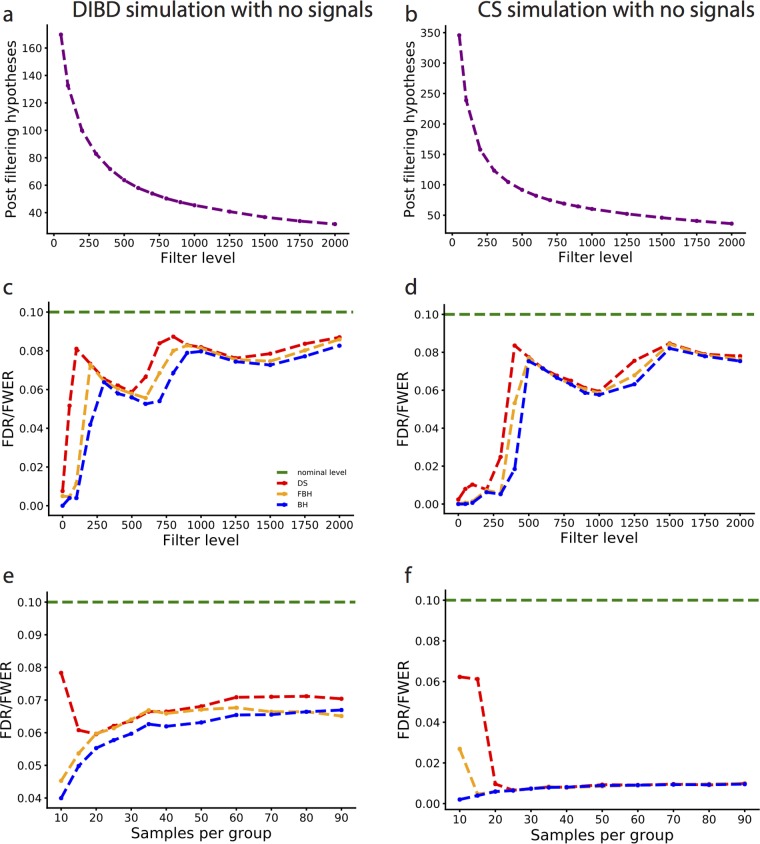
Comparison of FDR methods on data-driven simulations with no signals. (a) Number of hypotheses remained after filtering in the DIBD simulation, where the initial (nonfiltered) number of hypotheses is 867, for 20 samples per group. (b) Number of hypotheses after filtering in the CS simulation, where the initial (nonfiltered) number of hypotheses is 2,817, for 20 samples per group. (c) Estimated FDR equals FWER for the DS, FBH, and BH procedures (red, orange, and blue lines, respectively) as a function of the filter level in the DIBD simulation (20 samples per group) in panel a. The green line indicates the nominal FDR control level (0.1). (d) Same as panel c for the CS simulation. (e) Estimated FDR equals FWER for the DS, FBH, and BH procedures (red, orange, and blue lines, respectively) as a function of the number of samples in each group in DIBD simulation (20 samples per group and filter level at 1,000). The green line indicates the nominal FDR control level (0.1). (f) Same as panel e for the CS simulation.

In the global null setting, we want to use data-driven simulations to demonstrate that the DS-FDR controls the FDR when there are in fact no differentially abundant taxa at all in the study. Under the global null setting, the FDR will be equal to the FWER, the probability of at least one false-positive result. To simulate the data, in each real data set, we choose *N* samples (the desired number of samples per group in the simulated data) from the same group, split them randomly into healthy and sick groups, apply the FDR methods at a prespecified level of 0.1, and then count the number of times of making at least one false-positive error. By randomly taking samples from the real data set, the dependence across taxa in the simulated data is the same as in the real data. We repeat each simulation a large number of times to calculate the average number of making at least one false-positive error, until the standard error is less than 0.001. In scenario I (Fig. 3a to d), we set the samples to be 20 in each group and then increase the filter level from 0 to 2,000, where we throw away taxa with a total abundance less than the desired threshold. In scenario II (Fig. 3e and f), we vary the sample size (from 10 to 90 samples in each group) with a fixed filter level of 1,000 in the simulated data to generate different discreteness levels. The results (Fig. 3c to f) show that all three methods safely control the FDR in this global null setting, i.e., the probability of at least one false-positive result is at most 0.1 when there are no differentially abundant taxa in the data set.

**FIG 4:**
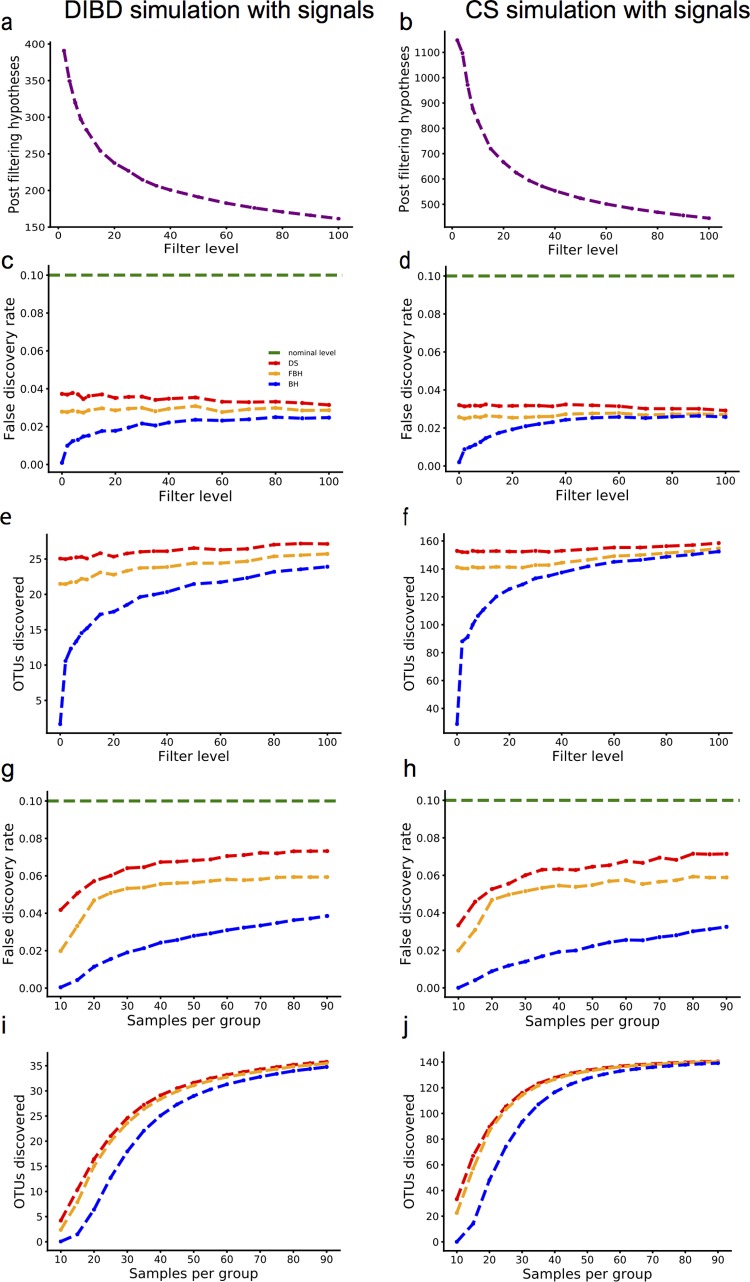
Comparison of FDR methods on data-driven simulations with signals. (a) Number of hypotheses after filtering in the DIBD simulation, where the initial (nonfiltered) number of hypotheses is 867, for 15 samples per group and 10% true signals. (b) Number of hypotheses after filtering in the CS simulation, where the initial (nonfiltered) number of hypotheses is 2,817, for 15 samples per group and 10% true signals. (c) Estimated FDR for the DS, FBH, and BH procedures (red, orange, and blue lines, respectively) as a function of filter level in the DIBD simulation (15 samples per group) in panel a. The green line indicates the nominal FDR control level (0.1). (d) Same as panel c for the CS simulation. (e) Number of truly differential OTU discoveries for the DS, FBH and BH procedures (red, orange, blue, respectively) as a function of filter level in panel a. (f) Same as panel e for the CS simulation. (g) Estimated FDR for the DS, FBH and BH procedures (red, orange, blue, respectively) as a function of number of samples in each group in the DIBD simulation (15 samples per group, filter level at 10). Green line indicates the nominal FDR control level (0.1). (h) Same as panel g for the CS simulation. (i) Number of truly differential OTU discoveries for the DS, FBH, and BH procedures (red, orange, and blue lines, respectively) as a function of number of samples in each group in panel g. (j) Same as panel i for the CS simulation.

Next, we introduce signals into the simulations. Similar to the global null setting, we choose *N* samples from the same group and then split them randomly into healthy and sick groups. Then, we add signals to a specific number of taxa to make them truly different between the healthy and sick groups (see supplemental material and Tables S2 and S3 for details). Finally, we apply the FDR methods at the prespecified level of 0.1 and calculate the average FDR and number of true signals detected by three methods. Each simulation was repeated for a large number of times until the standard error is less than 0.001. In scenario I (Fig. 4a to f), we set the sample size to be 15 in each group, the proportion of true signals at 10%, and the filter level varying from 0 to 100. In scenario II (Fig. 4g to j), we vary the sample size (from 10 to 90 samples in each group) at the fixed filter level of 10 and the proportion of true signals at 10%. The results (Fig. 4c to j) show that all methods control the FDR below the threshold (Fig. 4c and d and Fig. 4g and h), with DS-FDR detecting the largest number of significantly different taxa across all conditions compared to the other two methods (Fig. 4e and f and Fig. 4i and j). Also, DS-FDR is least affected by the severe discreteness shown in low filter level and small sample size. As the filter level or sample size increases (Fig. 4e and f and Fig. 4i and j), the simulated data become less discrete, and the other two methods are able to catch up with DS-FDR at a high filter level or large sample size, which coincides with previous simulation results in Fig. 2b and d.

10.1128/mSystems.00092-17.5 TABLE S2  Choice of *K* in the DIBD simulation with signals. Download TABLE S2, DOCX file, 0.1 MB. Copyright © 2017 Jiang et al. 2017 Jiang et al. This content is distributed under the terms of the Creative Commons Attribution 4.0 International license.

10.1128/mSystems.00092-17.6 TABLE S3  Choice of *K* in the CS simulation with signals. Download TABLE S3, DOCX file, 0.1 MB. Copyright © 2017 Jiang et al. 2017 Jiang et al. This content is distributed under the terms of the Creative Commons Attribution 4.0 International license.

We are interested in the procedure that maximizes the expected number of true discoveries, while still controlling the FDR at level at most *q*. Our empirical studies show that all the procedures considered have FDR at most *q*. Therefore, among all procedures considered, we would like to select the procedure with the greatest average number of true discoveries. This procedure is clearly DS-FDR. This is our preferred procedure despite the fact that the FDR level of DS-FDR is higher than that of the competing methods, since it is still below the nominal level we are willing to tolerate.

### Real data applications.

To validate the advantage of DS-FDR over the classical BH procedure in microbiome studies, we compared the three FDR methods using the following nine real microbiome data sets: CFS (gut microbiome in individuals with chronic fatigue syndrome) (9), MLT (gut microbiota in mice lacking Toll-like receptor 5 [TLR5]) (10), DME (delivery mode shapes the initial microbiota in newborns) (11), CD (gut microbiome in new-onset Crohn’s disease) (12), UKT (human fecal microbiome in the TwinsUK cohort) (13), DIBD (gut microbiome in dog and human inflammatory bowel disease) (7), CS (microbial communities in the upper respiratory tract of cigarette smokers) (8), AGP (American Gut Plant Number subset) (The American Gut Project), and AGA (American Gut Antibiotic History subset) (The American Gut Project). These data sets have different characteristics of microbiome data, covering a wide range of sample size, total number of taxa, and sparsity rate (number of nonzero entries in the entire data set) (Table S4). We will demonstrate how DS-FDR can help alleviate the effect of arbitrary filtering in microbiome analysis and its advantage in identifying similar numbers of differentially abundant taxa as the BH procedure does, yet with much smaller sample size.

10.1128/mSystems.00092-17.7 TABLE S4  Features of nine real microbiome data sets. Download TABLE S4, DOCX file, 0.05 MB. Copyright © 2017 Jiang et al. 2017 Jiang et al. This content is distributed under the terms of the Creative Commons Attribution 4.0 International license.

### Application I: alleviating the effect of arbitrary filtering.

**FIG 5:**
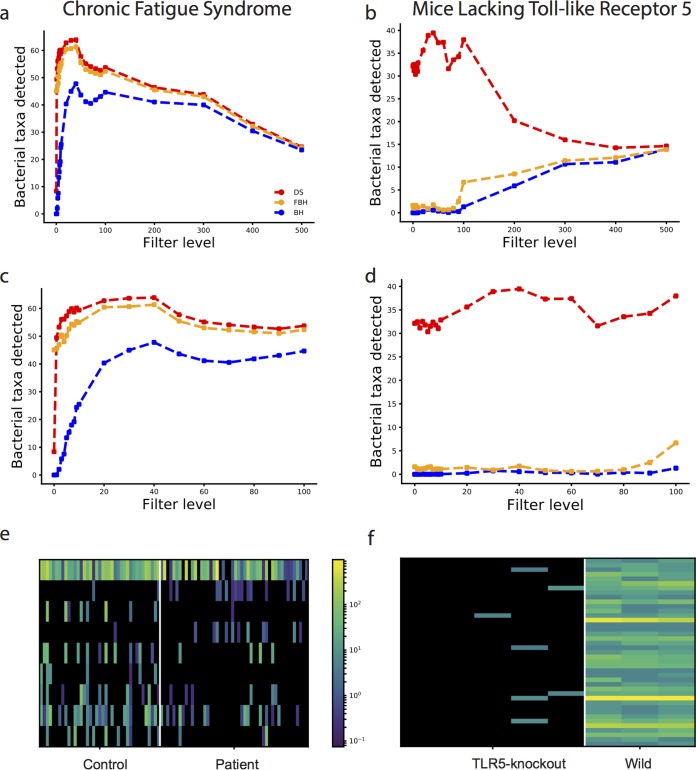
Performance of FDR methods on filtered microbiome data. (a) Number of differentially abundant taxa detected as a function of the taxon filtering level based on the CFS data set. Results for the DS, FBH, and BH procedures using an FDR threshold of 0.1 are shown by red, orange, and blue lines, respectively. (b) Number of differentially abundant taxa detected as a function of the taxon filtering level based on the MLT data set. Results for the DS, FBH, and BH procedures using an FDR threshold of 0.1 are shown by red, orange, and blue lines, respectively. (c) Expanded view of panel a for a filtering level of 0 to 100 on the CFS data set. (d) Expanded view of panel b for a filtering level of 0 to 100 on the MLT data set. (e) Heatmap displaying the additional taxa detected with DS-FDR (filtering threshold of 5) compared to FBH in CFS data. CFS data for healthy controls and patients are shown. Each row represents a unique taxon, and each column depicts a sample, with colors showing the relative abundance. Taxa are sorted according to the value of the statistic used (mean rank difference). (f) Same as panel e but for the MLT data. MLT data for the TL5 knockout mice and wild-type mice are shown.

One common dilemma in initial processing of microbial abundance tables is choosing the proper parameters to clean up the data. In standard experiments, many external factors introduce noise into the data set, such as contamination or sequencing error. These errors, together with real, rare microbes present only in a small fraction of the samples, are not informative for studying the variables of interest, and they reduce our ability to identify differential abundance because of the additional multiple-hypothesis testing burden they introduce. A common approach to overcome this problem is to filter out low-abundance taxa (i.e., taxa with total prevalence or abundance less than some desired threshold). However, this filtering can also remove biologically relevant taxa. Therefore, choosing the correct threshold is important for differential abundance testing. Figure 5a to d (blue line) shows the effect of minimal abundance filtering on the number of significant differential taxa detected using BH.

To demonstrate how DS-FDR can alleviate the effect of arbitrary filtering, we compared the FDR methods in the following two experiments on chronic fatigue syndrome (CFS) and mice lacking Toll-like receptor 5 (MLT). In both data sets, we drop samples with reads of less than 1,000 in each taxon. This is done to alleviate the possible effects of technical artifacts on our analysis. Hence, our CFS data set has 5,812 taxa present in 87 samples for 48 patients and 39 control participants, and the MLT data set contains 908 taxa in 5 TLR5 knockout mice and 3 wild-type mice. These two data sets were chosen because they display two different characteristics that can possibly cause discrete *P* values: large number of rare taxa or small sample size. The CFS data set is sparse with only 5.16% nonzero entries, whereas the MLT data set, which has about 33% nonzero values, has no more than five samples in each group.

As can be seen in Fig. 5a and b, low (or no) filtering can lead to a highly reduced detection power, whereas a high filtering level again can reduce the power (probably due to filtering of real relevant taxa). Due to the discrete nature of low-abundance taxa, DS-FDR can naturally alleviate this problem, thus enabling higher power even at low filtering levels (Fig. 5a to d, red lines), while reducing the need for arbitrary filtering criteria. Additionally, DS-FDR consistently detects more taxa as significantly different in both data sets. This is due to the fact that DS-FDR is a less conservative method compared to the other two methods. The extent of the increased number of significant taxa identified by DS-FDR compared to the other methods varies and depends on the underlying distribution of microbes and the number of samples. For example, in Fig. 5b, DS-FDR detects up to 40 taxa that are different between TLR5 knockout mice and wild-type mice, whereas BH and FBH detect between 3 (when no filtering is applied) and 15 (at higher filtering level) taxa. Additionally, note that while the performance of BH and FBH improves at higher filtering levels (due to removal of low-abundance taxa, which leads to a smaller number of hypotheses), DS-FDR shows a decrease in the number of taxa detected at higher filtering levels, indicating that many differentially abundant taxa have low abundance, and thus are thrown away when the filtering is applied. When investigating CFS instead, the increase in detected differentially abundant microbes between different methods is much less pronounced, probably because the discreteness problem is less severe (making this factor easier to pick up by less powerful methods).

To further validate that DS-FDR is detecting truly differential taxa, we show in Fig. 5e and f heatmaps of additional differentially abundant taxa detected by DS-FDR compared to FBH (using a filtering threshold of 5) for the two experiments. The significantly differential taxa found by DS-FDR in MLT are highly abundant in wild-type mice yet hardly exist in TLR5 knockout mice (Fig. 5f). Figure 5e also shows some taxa high in the controls while low in the patients or vice versa. This clear distinction between the two groups in both data sets indicates that DS-FDR is finding potentially interesting bacteria that merit further investigation.

In summary, filtering low-abundance taxa is necessary if performing BH; however, this procedure incurs the price of losing interesting behavior in the low-abundance taxa and introducing an arbitrary threshold that is typically not empirically justified. Using FBH partially solves this problem by removing taxa that cannot reach the minimal *P* value. However, DS-FDR requires neither this arbitrary threshold in BH nor the partially subjective filtering criterion in FBH, and it is much less sensitive to the filtering level, allowing the researcher to keep more taxa while paying a small price for multiple testing on the rare taxa.

### Application II: reducing the cost of additional samples in differential abundance identification.

**FIG 6:**
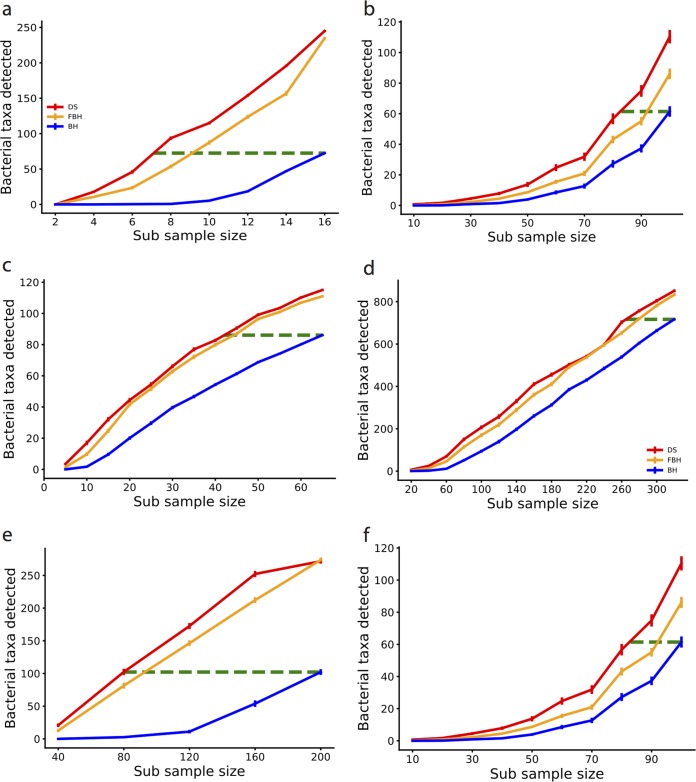
Performance of FDR methods on subsampling of microbiome data. (a) Number of differentially abundant taxa detected as a function of the sample size in each group on the DME data set. Results for the DS, FBH, and BH procedures using an FDR threshold of 0.1 are shown by red, orange, and blue lines, respectively. The green line represents the maximum number of differentially abundant taxa identified by BH, and the length of the green line shows the samples saved by DS-FDR for identifying similar number of taxa compared to the other two methods. (b) Same as panel a on the UKT data set. (c) Same as panel a on the DIBD data set. (d) Same as panel on the CD data set. (e) Same as panel a on the AGP data set. (f) Same as panel on the AGA data set.

DS-FDR has more statistical power than BH and FBH when the test statistics have different discrete null distributions. Therefore, fewer samples are required to identify differentially abundant microbes than when using the other two methods. This pattern is consistent across multiple experiments, including comparing vaginal births versus Caesarean section babies (Fig. 6a), obese versus lean twins (Fig. 6b), healthy dogs versus dogs with IBD (inflammatory bowel disease) (Fig. 6c), healthy subjects versus Crohn’s disease patients (Fig. 6d), subjects eating more than 30 types of plants per week versus eating less than 5 types (Fig. 6e), and subjects who have not taken antibiotics in the past year versus subjects who have taken antibiotics within a week (Fig. 6f). Figure 6 and Table 1 demonstrate that DS-FDR can detect the same number of bacterial OTUs with at least 15% fewer samples than needed for BH. However, the amount of improvement varies across these studies, ranging from a decent amount of 16% (CD data set) to as high as 60% (AGA data set) compared to BH and only 3% (AGP data set) to 22% (DME data set) compared to FBH. This could be due to a number of factors, such as the underlying microbial distribution and sparsity and the effect size of the sample groups. Note that a high reduction in sample size for DS-FDR compared to BH does not necessarily indicate that such a huge gap still exists between DS-FDR and FBH. For example, DS-FDR could use 52% fewer samples than BH on the AGP data set yet only 3% lower than FBH. The relatively close result of FBH to DS-FDR occurs in DIBD (7%), CD (4%), and AGP (3%) data sets, which share similar characteristics of having high sample size and low sparsity. This indicates that FBH could be competitive with DS-FDR in data sets with such discrete features. Overall, DS-FDR is shown to require the smallest number of samples to detect the same effect, which can reduce the cost of additional samples required for differential abundance identification.

**TABLE 1:** Summary of subsampling comparisons in Fig. 6^a^

Data set	No. of bacterial taxa detected	% fewer samples (DS vs BH)	% fewer samples (DS vs FBH)
DME	72	56	22
UKT	61	17	11
DIBD	86	35	7
CD	717	16	4
AGP	215	52	3
AGA	102	60	11

a DS, discrete-FDR method; BH, Benjamini-Hochberg FDR method; FBH, filtered Benjamini-Hochberg FDR method.

### Conclusion.

In both simulations and real data sets, we have shown that DS-FDR improves the power of statistical tests, especially compared to the traditional BH procedure, while controlling the FDR at the desired level. This has important practical consequences, such as alleviating the effects of arbitrary filtering in microbiome experiments or halving the required sample size for a given level of statistical power to detect differences in some cases. We note that this improvement is achieved although no additional assumptions are used in DS-FDR compared to BH or FBH. Moreover, future work can be done to exploit the dependency between tests, such as incorporating existing information about the grouped or hierarchical dependence between hypotheses under consideration when controlling the FDR (14, 15).

The application of DS-FDR is not limited to the multiple testing at the taxon level as mostly shown in this study; tests at higher taxonomic rank, such as family or phylum, are also applicable, although the advantage may be smaller in this case because fewer tests are performed and the data are less sparse. Because metabolomic data share similar characteristics of small sample size and sparsity with microbiome data sets, DS-FDR could also be applied to these and other omic data sets to improve the multiple testing procedures more generally. Moreover, DS-FDR can be combined with compositional tools, such as balances (16), ANCOM (17), and SparCC (18) to alleviate the compositional problem in numerous microbiome data analysis tasks.

## MATERIALS AND METHODS

In the BH procedure, how close the FDR control is to the nominal level depends critically on the distribution of *P* values under the null hypothesis. If a null hypothesis is simple (uniquely specifies the population distribution) and the corresponding test statistics are continuous, then the *P* values are uniformly distributed in [0, 1] under the null hypothesis, and therefore, the FDR is controlled at the exact level $\frac{m_{0}}{m}q$. However, if the test statistics are discrete, the null distribution of the *P* values is stochastically larger than the uniform distribution, and thus, the FDR could be much smaller than $\frac{m_{0}}{m}q$. This is because the expression for the FDR of the BH procedure involves sums with the term ${\mathrm{Pr}}_{H_{i}}\left(p_{i}\leq \frac{k}{m}q\right)$, where ${\mathrm{Pr}}_{H_{i}}$ indicates the probability computed under the true null hypothesis *H*_*i*_, *p*_*i*_ is the *P* value of a true null hypothesis *H*_*i*_, and *k* = 1, …, *m*. If the null distribution of the *P* value is uniform, then ${\mathrm{Pr}}_{H_{i}}\left(p_{i}\leq \frac{k}{m}q\;\right)=\frac{k}{m}q$. However, for the discrete test statistics, ${\mathrm{Pr}}_{H_{i}}\left(p_{i}\leq \frac{k}{m}q\right)$ may be less than $\frac{k}{m}q$, and the greater the gap between them, the smaller the true FDR level of the BH procedure. Thus, the BH procedure can be conservative for discrete test statistics, in the sense that its actual FDR level may be smaller than $\frac{m_{0}}{m}q$. Moreover, such conservatism does not decrease as the number of hypotheses increases or with modifications of the original BH procedure that can provide higher power by incorporating an estimate of the number of null hypotheses (19).

Several other approaches that take the discreteness into account for FDR control have been suggested in the literature. Kulinskaya and Lewin (20) suggested an FDR-controlling procedure using randomized *P* values to account for the discreteness of the null distribution, thus guaranteeing that the *P* values are uniformly distributed under the null hypothesis and that the FDR is controlled exactly at the desired level when the *P* values are independent. However, due to the randomness of the *P* values, interpretation of the results is not straightforward. Gilbert (6) proposed a two-step FDR-controlling procedure for discrete data. First, remove the null hypotheses with test statistics that are unable to reach the level of significance necessary if in the second step the Bonferroni procedure is applied, as suggested by Tarone (21). Second, apply the BH procedure to the remaining hypotheses. Although this approach reduces the essential dimensionality of the multiplicity problem and therefore can be more powerful than the BH procedure on all hypotheses, it does not exploit the discreteness of the test statistics that are not removed in the first step. Recognizing this limitation, Gilbert suggested that the BH procedure at level q* > *q* should be applied in the second step. However, there is no theoretical guarantee that the FDR is controlled (6), even if the *P* values are independent. Another limitation of Gilbert’s procedure is that the filtering step, which uses a Bonferroni-like threshold, may be too aggressive, as it filters out hypotheses for which the *P* value cannot reach a lower bound which is potentially much lower than the actual *P* value threshold using the BH step at the second step.

Motivated by the formulation of the BH procedure, Heyse (4) suggested a discrete BH procedure that exploits more fully the discrete null distributions of the test statistics and demonstrated in simulations that it controls the FDR at the prespecified level and has power equal to or greater than both the BH and Gilbert methods. Let *p*_(1)_ ≤ … ≤ *p*_(*m*)_ be the sorted *P* values (subscript parentheses indicate sorted *P* values, and raw *P* values are without parentheses), then the BH-adjusted *P* values are $p_{\left(j\right)}^{\text{BHad}j}={\text{min}}_{i\;\geq j}\;\frac{m}{i}p_{\left(i\right)}$, and the BH procedure at level *q* is equivalent to rejecting all hypotheses with BH-adjusted *P* values of ≤*q*. Heyse suggested a procedure that adjusts *P* values as $p_{\left(j\right)}^{\text{Heyse.adj}}=\text{min}\left(p_{\left(j+1\right)}^{\text{BHadj}},\;\frac{\sum_{l=1}^{m}{\text{Pr}}_{H_{l}}\;\left(p_{l}\;\leq p_{\left(j\right)}\right)}{j}\right)$, and the procedure at level *q* is equivalent to rejecting all hypotheses with Heyse-adjusted *P* values of ≤*q*. The procedure by Li and Tibshirani (5) can be expressed as the DS-FDR procedure that adjusts *P* values as $p_{\left(j\right)}^{\text{DS-FDR.adj}}={\text{min}}_{i\geq j}\frac{\sum_{l=1}^{m}{\text{Pr}}_{H_{l}}\;(p_{l}\;\leq \;p_{\left(i\right)})}{i}$, where the null probabilities are estimated using permutations of the labels (see supplemental material for the proof and see the detailed algorithm below). These adjusted *P* values are at least as small as Heyse’s adjusted *P* values (when the probabilities are computed by permutations); thus, the DS-FDR procedure is potentially more powerful than Heyse’s procedure. The gain from using this procedure over the BH procedure comes from the fact that ${\text{Pr}}_{H_{l}}\;\left(p_{l}\;\leq p_{\left(i\right)}\right)\leq p_{\left(i\right)}$. If hypothesis *H*_*l*_ cannot achieve a *P* value below *p*_(*i*)_, then ${\text{Pr}}_{H_{l}}\left(p_{l}\leq \;p_{\left(i\right)}\right)=0$ and the dimensionality of the multiple comparisons problem is reduced. If hypothesis *H*_*l*_ can achieve a *P* value below but not equal to *p*_(*i*)_, then ${\text{Pr}}_{H_{l}}(p_{l}\leq p_{\left(i\right)})\;<\;p_{\left(i\right)}$ and a smaller quantity adds to $P_{\left(j\right)}^{\text{DS-FDR.adj}}$. On the other hand, if all the null distributions are identical, then there is no gain in using the DS-FDR procedure over the original BH procedure. Therefore, DS-FDR procedure rejects at least as many null hypotheses as the BH procedure. However, if all null distributions are the same, then the DS-FDR procedure rejects exactly the same null hypotheses as the BH procedure does.

In microbiome data, the null distribution of test statistics is unknown, and therefore, it is often inappropriate to make parametric assumptions. The discrete (DS) FDR procedure, an application of Li and Tibshirani’s FDR method (5) to microbiome data analysis, generates the null distribution of the statistic using permutations, and therefore is potentially very useful. Even though Li and Tibshirani’s FDR method was not developed specifically for discrete test statistics, we find that it yields a useful procedure for discrete data. The DS-FDR algorithm follows.

1. Compute the test statistics for the original labeling of observations from *m* taxa *T*_1_,…,*T_m_*.
2. Permute the labels *B* times, and recalculate all the test statistics each time. In the *b*th permutation, denote the computed statistics $T_{j}^{*b},j=1,\;.\;.\;.,m$.
3. For a range of values of the cut-point *C*, compute V^=∑j=1m∑b=1BI ( |Tj*b| ≥ C)+I (|Tj| ≥C)B+1 and R^ =∑j=1mI (|Tj|≥C).
4. Estimate the FDR at the cut-point *C* by FDRc ^= V^R^.
5. Find $\hat{C}=\arg{\text{min}}_{c}\{\;\hat{{\text{FDR}}_{c}\;}\leq q\}$. If no solution exists, set *Ĉ* = infinity.
6. Reject all hypotheses with |*T_j_*| ≥ *Ĉ*.

Here we compare the performance of DS-FDR with the BH procedure and the filtered BH (FBH) procedure on simulated and real microbiome data. The FBH procedure is analogous to Gilbert’s (6) two-step procedure, and it is preferred over Gilbert’s method as it is theoretically guaranteed that the FDR is controlled and is less aggressive in removing potentially interesting taxa: first, reduce the number of tests by eliminating hypotheses whose minimal achievable *P* value cannot reach the nominal unadjusted *q* level of statistical significance, and second, apply the BH procedure to the reduced set of microbes. In the first step, we calculate the smallest *P* value achievable for each hypothesis test, determined by computing a *P* value for the most extreme possibility that all of the observed responses occurred in the same group, and then remove those microbes whose corresponding minimal *P* value cannot reach the prespecified *q* level. This approach reduces the dimensions of multiple testing and increases our power to detect differentially abundant microbes. As shown in the simulations (Fig. 2 to 4), FBH alleviates the conservatism in BH procedure, but it is still inferior to the DS-FDR procedure, because FBH does not exploit the discreteness of the test statistics that are not removed in the first step. Therefore, we recommend the DS-FDR, since it has the advantage of not deteriorating in handling discrete data and not requiring any type of predefined filtering threshold like FBH to reduce the number of hypotheses. We expect that DS-FDR will be a valuable tool for microbiome analysis to discover additional microbes that would not be discovered by the BH and FBH procedures. Since further investigations on these additional microbes may be costly, it is important not to launch investigations into too many false leads. We have shown in simulations and applications to real data that the DS-FDR procedure has higher power to discover truly differentially abundant microbes than other FDR-controlling procedures, while guaranteeing that only a well-defined fraction of the discovered associations are false-positive results.

To facilitate its adoption by the microbiome community, DS-FDR is available in Calour (https://github.com/biocore/calour), an exploratory and interactive tool for microbiome analysis, as well as a standalone python module (https://github.com/biocore/dsfdr) and a QIIME-2 plugin (https://github.com/serenejiang/q2_dsfdr). Also, the Ipython notebooks and python scripts used to perform all the analyses and simulations can be found in https://github.com/knightlab-analyses/dsfdr-analyses.
