# Supplementary material for: Discrete False-Discovery Rate Improves Identification of Differentially Abundant Microbes
Source: mSystems. 2017 Nov 21;2(6):e00092-17. doi: 10.1128/mSystems.00092-17 (PMC5698492; doi:10.1128/mSystems.00092-17)
Supplement: TEXT S1 [file sys006172152s7.docx]

**Simulation Details**

In order to approximate a microbiome experiment, the simulation contains two groups of samples (Sick and Healthy) with taxa from three different categories. In the first category, taxa originate from a normal distribution with different means for the Sick and Healthy groups (the mean for the Sick group was chosen randomly from a uniform distribution U(3, 4), and the mean for the Healthy group was chosen randomly from a uniform distribution U(5, 6), and the standard deviation is the same for both groups as 2), and thus the null hypotheses that taxa in the two groups come from the same distribution are incorrect. In the second category, we simulate taxa unaffected by the Sick or Healthy groups. Taxa are drawn from a single normal distribution for all samples (mean was chosen randomly from a uniform distribution U(20, 21) and standard deviation as 2), therefore the null hypotheses are true for them. In the third category, we introduce a set of rare taxa. These taxa are present in a very small number of samples, and are created by randomly choosing one to six non-zero samples per bacterium. Table S1 summarizes the setting of our simulated data.

In simulation 1, we test the effect of sample size on different FDR procedures. Hence, we simulate 100 truly different taxa (Category I), 100 taxa coming from the same distribution (Category II), and 800 rare taxa (Category III). Sample size varies from 10 to 100 per group, the difference of the mean rank is used as the test statistic, q = 0.1 is chosen as the threshold for FDR, and 1000 permutations are performed in FDR calculation.

In simulation 2, we check the effect of sparsity on FDR methods. Thus, we choose the sample size to be 50 in each group to reduce the discrete effect resulting from small sample size, and we simulate 100 truly different taxa (Category I) and 100 taxa from the same distribution (Category II), yet varying the number of rare taxa from 500 to 10,000 (Category III). The choice of test statistics, nominal FDR level and number of permutations are the same as in simulation 1.

**Data Driven Simulations Details**

Global null simulation:

Steps:

1. Normalize the original data so that each sample will have total reads of 10000.
2. Choose one group from the normalized data, let the sample size be M and number of taxa be p (‘filtering’ added at this step for scenario II when filter level is fixed but sample size varies).
3. Choose N subjects to be ‘healthy’ out of M, and another N to be ‘sick’ (sampling with replacement, N <= M).
4. Filtering the simulated data for scenario I when sample size is fixed but filter level varies.
5. Apply FDR methods to the simulated data.

Simulation with signals:

Steps:

1. Normalize the original data so that each sample will have total reads of 10000.
2. Choose one group from the normalized data, let the sample size be M and number of taxa be p (‘filtering’ added at this step for scenario II when filter level is fixed but sample size varies).
3. Choose N subjects to be ‘healthy’ out of M (sampling with replacement, N <= M), and N subjects to be ‘sick’.
4. Specify $p_{1}$, the number of taxa with real signal, then $p_{0}=p- p_{1}$is the number of true nulls, and c = p1/p, the proportion of truly differentially abundant taxa.
5. The first half $p_{1}$ taxa in ‘healthy’ + Normal ($\mu_{H}$, $\sigma$);

the first half $p_{1}$ taxa in ‘sick’ + Normal ($\mu_{S}$, $\sigma$);

the second half $p_{1}$ taxa in ‘healthy’ + Normal ($\mu_{S}$, $\sigma$);

the second half $p_{1}$ taxa in ‘sick’ + Normal ($\mu_{H}$, $\sigma$).

1. Filtering the simulated data for scenario I when sample size is fixed but filter level varies.
2. Apply FDR methods to the simulated data.

Note: let k be the difference between $\mu_{H}$ and $\mu_{S}$ (see table S3 and S4 for choice of K).

In the simulations: $\mu_{H}$~ uniform (5, 6), k = 2, $\mu_{S}$= $\mu_{H}$ + k, $\sigma$ = 2, c = 10%.

**Proof** **of the equivalence of DS-FDR adjusted p-value to DS-FDR algorithm**

To see the equivalence between DS-FDR adjusted p-value and the DS-FDR algorithm, we need to show that the set of hypotheses with $p_{(j)}^{DS-FDR.adj} \leq q$ is equivalent to the set of hypotheses with $\left| T_{j} \right|\geq\hat{C}$ in the DS-FDR algorithm.

With a slight abuse of notation, we treat the p-value computation as exact even though the p-values are computed based on B permutations, so $p_{j}= \frac{\sum_{b=1}^{B} I \left( \left| T_{j}^{*b} \right|\geq\left| T_{j} \right| \right)+1}{B+1}$.

Then

$$\Pr(p_{l} \leq p_{i})= \frac{\sum_{b=1}^{B} I \left( \left| T_{l}^{*b} \right|\geq\left| T_{i} \right| \right)+I \left( \left| T_{l} \right|\geq\left| T_{i} \right| \right)}{B+1}.$$

Since $\left| T_{\left( 1 \right)} \right| \geq\ldots\geq\left| T_{\left( m \right)} \right|,$

$$p_{(j)}^{DS-FDR.adj}= {min}_{i \geq j} \frac{\sum_{l=1}^{m} \frac{\sum_{b=1}^{B} I \left( \left| T_{l}^{*b} \right|\geq\left| T_{(i)} \right| \right)+I (\left| T_{l} \right|\geq\left| T_{(i)} \right|)}{B+1}}{i}.$$

If there exists a solution for $\hat{C}$, clearly it is enough to limit $\hat{C}$ to be one of the |$T_{(1)}|, \ldots, |T_{\left( m \right)}|$. Suppose $j^{*}$ is the value such that $\hat{C}=\left| T_{\left( j* \right)} \right|$. If $j < j^{*}$, then the hypothesis corresponding to $T_{(j)}$ is rejected according to the DS-FDR algorithm. It is also rejected based on the DS-FDR adjusted p-value, since $p_{(j)}^{DS-FDR.adj} \leq\frac{\sum_{l=1}^{m} \frac{\sum_{b=1}^{B} I \left( \left| T_{l}^{*b} \right|\geq\left| T_{(j*)} \right| \right)+I (\left| T_{l} \right|\geq\left| T_{(j*)} \right|)}{B+1}}{j^{*}} \leq q$.

If $j> j^{*}$, then the hypothesis corresponding to $T_{(j)}$ is not rejected according to the DS-FDR algorithm. Since from the definition of $j^{*}$ it follows that for $i> j^{*}$, then

$\hat{{FDR}_{T_{(i)}}}= \frac{\frac{\sum_{l=1}^{m} \sum_{b=1}^{B} I \left( \left| T_{i}^{*b} \right|\geq\left| T_{\left( i \right)} \right| \right)+I ( \left| T_{l} \right|\geq\left| T_{\left( i \right)} \right|)}{B+1}}{i} >q$, it follows that $p_{(j)}^{DS-FDR.adj} >q$.

Since every j is either rejected or not rejected by both procedures, the proof is complete.

Note that if there does not exist a solution for $\hat{C}$, i.e. the estimated FDR is above q for every possible value, then there is no rejection and $p_{(j)}^{DS-FDR.adj} >q$ for all $j$.
