# Supplementary material for: Discrete False-Discovery Rate Improves Identification of Differentially Abundant Microbes
Source: mSystems. 2017 Nov 21;2(6):e00092-17. doi: 10.1128/mSystems.00092-17 (PMC5698492; doi:10.1128/mSystems.00092-17)
Supplement: TABLE S1 [file sys006172152st3.docx]

**Table S1: Setting of simulated microbiome communities**

| Bacteria | Null Hypotheses | Group Healthy | Group Sick |
| --- | --- | --- | --- |
| Category I: different between groups | Incorrect | H1 | S1 |
| Category II: same distribution in both groups | Correct | H2 = S2 | |
| Category III: rare taxa (1-6 nonzero samples) | Correct | H3 = S3 | |
