# Supplementary figures and images for: Discrete False-Discovery Rate Improves Identification of Differentially Abundant Microbes
Source: mSystems. 2017 Nov 21;2(6):e00092-17. doi: 10.1128/mSystems.00092-17 (PMC5698492; doi:10.1128/mSystems.00092-17)

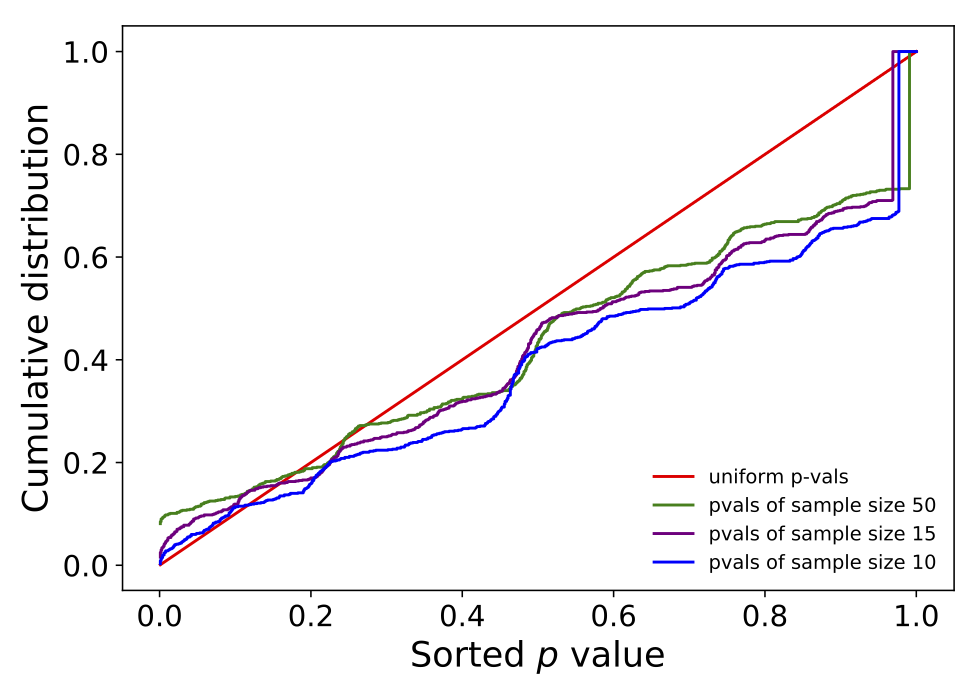

Supplement: FIG S1 [file sys006172152sf1.tif]

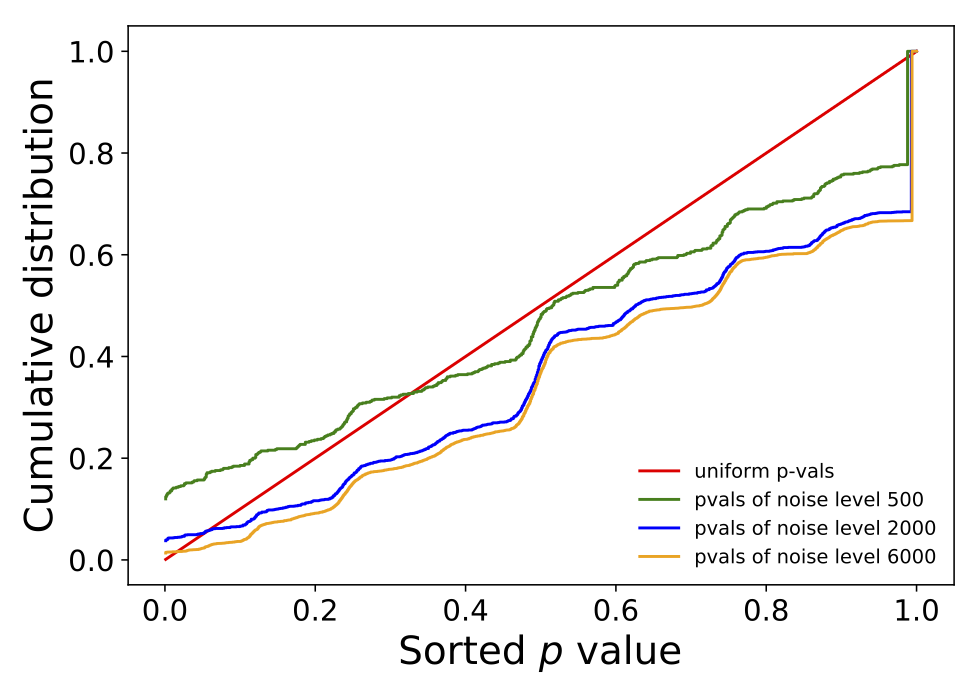

Supplement: FIG S2 [file sys006172152sf2.tif]
