# Supplementary material for: Discrete False-Discovery Rate Improves Identification of Differentially Abundant Microbes
Source: mSystems. 2017 Nov 21;2(6):e00092-17. doi: 10.1128/mSystems.00092-17 (PMC5698492; doi:10.1128/mSystems.00092-17)
Supplement: TABLE S2 [file sys006172152st4.docx]

**Table S2:** choice of K in DIBD simulation with signals

| DIBD simulation with signals | | |
| --- | --- | --- |
| Scale of K | FDR (BH/FBH/DS) | Power (BH/FBH/DS) |
| 1 | 0.0027/0.0123/0.0171 | 0.0017/0.0059/0.0093 |
| 2 | 0.0160/0.0288/0.0366 | 0.1725/0.2535/0.2868 |
| 3 | 0.0194/0.0323/0.0384 | 0.5217/0.5766/0.5941 |
| 4 | 0.0196/0.0325/0.0389 | 0.6549/0.6853/0.6944 |
| 5 | 0.0202/0.0340/0.0409 | 0.7017/0.7270/0.7340 |
| 6 | 0.0199/0.0337/0.0403 | 0.7250/0.7482/0.7545 |
| 7 | 0.0203/0.0346/0.0412 | 0.7410/0.7629/0.7688 |
| 8 | 0.0200/0.0344/0.0411 | 0.7527/0.7740/0.7792 |
| 9 | 0.0203/0.0348/0.0410 | 0.7616/0.7815/0.7865 |
| 10 | 0.0199/0.0346/0.0410 | 0.7693/0.7885/0.7934 |
| 20 | 0.0202/0.0356/0.0415 | 0.8180/0.8347/0.8387 |
| 30 | 0.0203/0.0361/0.0419 | 0.8460/0.8606/0.8638 |
| 40 | 0.0203/0.0363/0.0418 | 0.8647/0.8778/0.8809 |
| 50 | 0.0200/0.0363/0.0414 | 0.8808/0.8925/0.8952 |
| 60 | 0.0199/0.0364/0.0417 | 0.8924/0.9028/0.9053 |
| 70 | 0.0206/0.0371/0.0422 | 0.9004/0.9101/0.9122 |
| 80 | 0.0202/0.0374/0.0427 | 0.9072/0.9160/0.9180 |
| 90 | 0.0201/0.0370/0.0418 | 0.9120/0.9204/0.9223 |
| 100 | 0.0207/0.0380/0.0427 | 0.9162/0.9243/0.9264 |
