# Supplementary material for: Discrete False-Discovery Rate Improves Identification of Differentially Abundant Microbes
Source: mSystems. 2017 Nov 21;2(6):e00092-17. doi: 10.1128/mSystems.00092-17 (PMC5698492; doi:10.1128/mSystems.00092-17)
Supplement: TABLE S3 [file sys006172152st5.docx]

**Table S3:** choice of K in CS simulation with signals

| CS simulation with signals | | |
| --- | --- | --- |
| Scale of K | FDR (BH/FBH/DS) | Power (BH/FBH/DS) |
| 1 | 0.0002/0.0380/0.0136 | 0.0005/0.0054/0.0166 |
| 2 | 0.0140/0.0255/0.0316 | 0.3919/0.5006/0.5414 |
| 3 | 0.0163/0.0315/0.0364 | 0.8127/0.8553/0.8657 |
| 4 | 0.0164/0.0320/0.0360 | 0.9166/0.9311/0.9348 |
| 5 | 0.0165/0.0319/0.0359 | 0.9403/0.9491/0.9514 |
| 6 | 0.0164/0.0319/0.0359 | 0.9493/0.9562/0.9581 |
| 7 | 0.0165/0.0321/0.0361 | 0.9545/0.9605/0.9622 |
| 8 | 0.0166/0.0322/0.0361 | 0.9578/0.9632/0.9647 |
| 9 | 0.0165/0.0320/0.0359 | 0.9606/0.9656/0.9670 |
| 10 | 0.0167/0.0322/0.0361 | 0.9626/0.9675/0.9689 |
| 20 | 0.0167/0.0321/0.0360 | 0.9734/0.9769/0.9779 |
| 30 | 0.0166/0.0319/0.0358 | 0.9787/0.9817/0.9825 |
| 40 | 0.0166/0.0319/0.0357 | 0.9824/0.9849/0.9856 |
| 50 | 0.0166/0.0320/0.0359 | 0.9851/0.9872/0.9878 |
| 60 | 0.0166/0.0320/0.0358 | 0.9870/0.9889/0.9894 |
| 70 | 0.0165/0.0318/0.0358 | 0.9884/0.9901/0.9906 |
| 80 | 0.0167/0.0322/0.0361 | 0.9895/0.9911/0.9916 |
| 90 | 0.0166/0.0318/0.0357 | 0.9903/0.9919/0.9923 |
| 100 | 0.0167/0.0319/0.0359 | 0.9912/0.9926/0.9930 |
