# Supplementary material for: Discrete False-Discovery Rate Improves Identification of Differentially Abundant Microbes
Source: mSystems. 2017 Nov 21;2(6):e00092-17. doi: 10.1128/mSystems.00092-17 (PMC5698492; doi:10.1128/mSystems.00092-17)
Supplement: TABLE S4 [file sys006172152st6.docx]

**Table S4**: features of nine real microbiome datasets

| Data Set | Sample Size  (Group 1 vs. Group 2) | Number of Bacteria | Sparsity  (% non-zero entries) |
| --- | --- | --- | --- |
| CFS | 39 vs. 48 | 5812 | 5.16 % |
| MLT | 5 vs. 3 | 908 | 32.71 % |
| DME | 27 vs. 17 | 3774 | 5.17 % |
| CD | 719 vs. 333 | 9511 | 4.06 % |
| UKT | 273 vs. 114 | 8480 | 12.39 % |
| DIBD | 89 vs. 69 | 867 | 7.31 % |
| CS | 125 vs. 110 | 2817 | 4.60 % |
| AGP | 434 vs. 270 | 37878 | 0.47 % |
| AGA | 853 vs. 126 | 37878 | 0.49 % |
